# Postoperative Supine Sleeping Position Following Total Knee Arthroplasty Decreases Knee Flexion Contractures

**DOI:** 10.51894/001c.123412

**Published:** 2024-09-09

**Authors:** Robert L. Zondervan, Patrick K. Riggle, Adam J. Cien, Philip C. Penny, Jason M. Cochran

**Affiliations:** 1 McLaren Greater Lansing Hospital, Lansing, MI, USA; 2 Sparrow Hospital, Lansing, MI, USA; 3 Michigan State University, East Lansing, MI, USA; 4 Michigan Orthopedic Center, Lansing, MI, USA

## Abstract

**Background:**

Total knee arthroplasty (TKA) is an orthopaedic operation that improves quality of life and reduces pain in patients with disabling arthritis of the knee. One commonly recognized postoperative complication is flexion contracture of the knee. While early physical therapy and range of motion (ROM) exercises have helped improve ROM postoperatively, flexion contractures still remain a significant postoperative complication of TKA.

This study evaluated postoperative sleeping position and its effect on terminal knee extension and ROM following primary TKA. We hypothesized that patients who slept in the supine position would achieve earlier knee extension and ROM when compared to those in the lateral recumbent position.

**Methods:**

A total of 150 consecutive primary total knee arthroplasties (TKAs) were conducted by a single surgeon (JMC). Prospective data collection included assessments of preoperative range of motion (ROM), postoperative ROM, patient-reported outcome measures, and sleeping positions. Functional outcomes and patient-reported measures were compared between pre- and postoperative phases, as well as across different sleeping position groups.

**Results:**

Postoperative follow up was a mean of 29.6 days. Mean postoperative terminal extension ROM at one month was 2.98 degrees in the supine group versus 6.03 degrees in the lateral group (P < 0.001). Overall, there was significant improvement in patient reported outcome measures (WOMAC, Oxford, and pain) after surgery, but no difference existed between sleeping groups. For knee extension, a two-way ANOVA revealed that there was a statistically significant interaction between the effects of surgery and sleep position (p = 0.0053).

**Conclusions:**

Our results demonstrate that sleeping position does affect initial postoperative knee terminal extension; however, there is no effect on patient reported outcomes. We found a statistically significant difference in extension when comparing patients in the supine versus lateral group. Patients who slept in the lateral position lacked 6.03 degrees of extension which is greater than the 5 degrees threshold needed for normal gait mechanics. Conversely, those in the supine group only lacked 2.98 degrees of extension, allowing for normal gait mechanics. This study identifies an easy, effective means of increasing patient knee range of motion following TKA.

## Introduction

Total knee arthroplasty (TKA) is considered a routine and successful orthopaedic operation that greatly improves quality of life and reduces pain in patients with disabling arthritis of the knee.^1^ TKA procedures are performed frequently in the United States with over 935,000 procedures expected to be performed each year by 2030; however, some patients have difficulty regaining motion in the knee and can go on to develop postoperative flexion contractures.^2,3^ In order to perform regular activities of daily living, Rowe et al. demonstrated that a knee flexion range of motion (ROM) of 110 degrees is necessary and many have advocated for this to be a rehabilitation goal following TKA.^4^ Decreased ROM postoperatively affects patients gait and mobility and can lead to other musculoskeletal problems including low back pain.^5^ It is important to emphasize with patients the importance of preserving ROM following surgery to avoid scar tissue from developing in the knee, which can permanently impede future flexibility.

Postoperative ROM studies have demonstrated flexion contractures are one of the most frequent complications of TKA, occurring in as many as 1.4% to 17% of patients.^6–9^ Flexion contractures after a TKA is a significant source of morbidity for patients and early emphasis is placed on preserving pre-operative ROM.^10^ Long term results of flexion contractures can cause altered biomechanics leading to pain. It has been found that even contractures as little as 5 degrees can cause a noticeable limp during ambulation.^11,12^ When a flexion contracture is present, the quadriceps muscle will compensate by increasing the forces needed to stabilize the flexed knee during weight bearing. This can cause straining of the quadriceps muscle as it attempts to stabilize the knee and contribute to patellofemoral pain and a painful limp.^13^ It has been well established that the most recognized risk factor for the development of a flexion contracture is pre-operative ROM. Patients with a preoperative flexion contracture as small as 5 degrees are at least 2.9 times more likely to develop a postoperative flexion contracture.^14^ This alone, however, does not preclude those individuals without preoperative flexion contracture from developing postoperative flexion contracture. While this complication is burdensome to the patient’s lifestyle, it also often leads to dissatisfaction following surgery and may lead to further surgical intervention such as manipulation under anesthesia and revision TKA. While only 9.3% of revision surgeries are performed for stiffness, the overall number of TKA surgeries and revisions continues to increase and is projected to continue to increase in prevalence.^15^ Considering these possibilities, care in the initial postoperative time period is of vital importance not only for increased patient satisfaction but to prevent costly additional surgeries.

Given the amount of time patients spend sleeping, it is believed that patients sleeping with their knee in a flexed position will counteract their work with physical therapy. Because of this, many orthopedic surgeons advocate that patients sleep in a supine position rather than a lateral recumbent position in order to keep their knees extended as opposed to flexed. Some even go so far as to advocate for sleeping in a knee immobilizer to help limit motion across the knee joint following TKA, especially in the initial postoperative period.^16^ Thus, the purpose of this study was to review the effect of patient reported sleeping position in the initial postoperative period following primary TKA. We hypothesized that patients who reported sleeping primarily in the supine position would achieve greater knee extension and ROM when compared to individuals who reported sleeping primarily in the lateral recumbent position.

## Materials and Methods

Data was collected from a total of 150 consecutive primary TKAs that were performed by a single surgeon (JMC). Institutional Review Board (IRB) approval was obtained prior to initiation of the study. Patients were excluded from analysis in the study if they scheduled but subsequently cancelled surgery, did not follow-up postoperatively, or underwent revision surgical intervention secondary to complications.

Prior to undergoing TKA, informed consent was obtained, and all patients were counseled thoroughly by the senior author about expectations and criteria for discharge, standardized postoperative institutional rehabilitation protocol, and standardized postoperative pain management protocol. This preoperative counseling protocol was standardized and did not deviate between patient groups. Preoperative ROM was assessed prospectively while postoperative ROM, key functional score assessments, and sleeping position were assessed at the first postoperative office visit. Patients were then divided into two groups based upon their self-reported sleeping position questionnaire administered at the first follow up visit; those who slept primarily in the supine position and those who slept primarily in the lateral recumbent position.

Following surgery, full weight bearing was permitted as tolerated in both groups on postoperative day (POD) zero. For patients who underwent surgery and arrived on the orthopaedic floor prior to midday, physical therapy began with a single session on POD zero. Patients received two 30-minute physical therapy treatment sessions per day, as tolerated. Physical therapy consisted of general therapeutic exercises including ROM and manual resistive exercises, bed mobility education, transfer training, and gait training with the use of a front wheel walker. Postoperative physical therapy and mobility exercises did not vary between the two study groups.

Patient discharge timing was standardized, as per our institutional protocol. Patients were discharged from the hospital once their pain was adequately controlled (pain score < 5 on VAS per institutional protocol) with oral analgesia and they were independent with physical therapy. Patients were considered able to ambulate independently once they could ambulate greater than or equal to 150 feet independently per our institutional protocol and based off of previous data published by Keith et al.^17^ Patients followed up at approximately one month postoperatively in the surgeons office.

At the one month follow up appointment, knee joint ROM was assessed using a goniometer with the patient in the supine position. Goniometric measurements were taken by the same physician, who was blinded to the patients’ sleeping position. Multiple studies have shown moderate to high intra- and inter-rater reliability for goniometric measurement of knee ROM, although inter-rater agreement tends to be lower. It is generally recommended that the same clinician perform all measurements when assessing ROM.^18–20^ Therefore, in order to improve reliability and reproducibility in measuring ROM, one physician took all measurements and the goniometer axis was consistently placed on the lateral epicondyle of the femur with the proximal arm parallel to the long axis of the femur, pointing at the greater trochanter. The distal arm was consistently placed parallel to the long axis of the fibula, pointing at the lateral malleolus.^18–20^

The primary outcome measures of this study included knee range of motion (flexion and extension), and patient reported outcome scores (UCLA, Oxford, Womac, and pain score) using the self-reported patient questionnaire administered at the first follow up appointment.^1,6^ The primary independent variables were surgical time (pre- versus postoperative) and sleep position (supine versus lateral). An *a priori* sample size calculation was used to calculate 97 patients would be needed to detect a 10% difference with a 95% confidence level. To account for loss to follow up and surgery cancelation, an enrollment size of 150 patients was chosen. Continuous dependent variables were tested for normalcy using the Shapiro-Wilk test. Normally distributed continuous variables were compared using independent or paired t-tests. Non-Normally distributed continuous variables were compared using Wilcoxon signed-ranked or Mann-Whitney U tests. Means and standard deviations or medians and interquartile range were calculated for normally and non-normally distributed continuous variables, respectively. Binomial categorical variables were reported as percentages and compared using chi-squared test. Mixed-effects analysis was performed on the impact of sleep position and surgery using a two-way ANOVA. Statistical analysis was performed using Prism 10.2.1 (GraphPad, Boston, MA). The statistical significance was set at *P* ≤ 0.05. For multiple comparisons, a significance level Bonferroni correction was used.

## Results

A total of 150 patients were enrolled in this study and divided into two groups: A supine sleeping position group (n=74) and a lateral recumbent sleeping position group (n=76). One patient was excluded from the study after developing an acute traumatic wound dehiscence on postoperative day seven and twelve patients were excluded after scheduling surgery but subsequently cancelling it. Nine patients were excluded for not following up after surgery. The exclusion of these subjects, including their preoperative data, did not result in any statistically significant difference in the end results. The final patient count included for analysis was n=61 patients in the supine sleeping position group and n=67 patients in the lateral recumbent sleeping position. The patient cohort was comprised of 37.5% females (48/127). The average patient age of patients was 64.88 (±10.18) years old, the average BMI was 34.84 (±6.9), and surgery was performed on 47.7% (61/127) right knees. Patients were seen in office for examination on day 29.57 (±3.5) after surgery.

**Figure 1.**
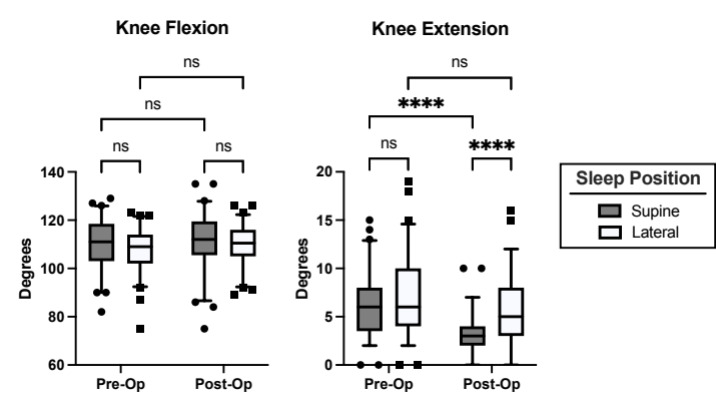
Pre- and postoperative knee range of motion between patients who sleep in the supine and lateral position. Box plots presented as median with 5-95 percentile. Pre-Op = Preoperative range of motion exam; Post-Op = Postoperative range of motion exam; ns = not significant; **** = P < 0.0001.

There were no statistically significant demographic differences found between the supine and lateral sleep position groups with regards to patient age, sex, BMI, POD, and operative side (Table 1). When combining supine and lateral groups, and looking at pre- versus postoperative changes, there was a significant improvement in knee extension, but not knee flexion (Table 2). There were also significant improvements in patient reported Oxford, WOMAC, and pain scores (Table 2). There was improvement in the UCLA score, but it did not reach statistical significance (p = 0.06). When comparing patient reported outcomes between supine and lateral sleeping position, there were no significant differences between groups in the pre- or postoperative period (Table 3). A two-way ANOVA was performed to analyze the effect of surgery and sleep position on knee range of motion. For knee extension, the two-way ANOVA revealed that there was a statistically significant interaction between the effects of surgery and sleep position (F (1, 125) = 8.040; p = 0.0053). Simple main effects analysis showed that surgery did have a statistically significant effect on knee extension (p = <0.0001). Additionally, simple main effects analysis of sleep position showed it had a statistically significant effect on knee extension (p = <0.0001). No significant interaction or simple main effect was found for the effect of surgery and sleep position on knee flexion. Paired comparisons of knee flexion and extension between and within the supine and lateral sleep groups were conducted in in the pre- and postoperative time points (Figure 1). There was a significant postoperative improvement in knee extension in the supine sleep group when compared to pre-operative supine scores and postoperative lateral scores. There were no significant differences in the lateral sleep groups.

**Table 1.** Patient demographic data.

	**Supine (n=61)**			**Lateral (n=67)**			
**Variable**	**Mean**	**SD**		**Mean**	**SD**	**Test**	**P**
Age (years)	65.39	9.848		64.4	10.52	t-Test	0.584
BMI (kg/m^2)	35.42	7.609		34.3	6.185	t-Test	0.363
Post-Operative Day	29.77	2.753		29.39	4.075	t-Test	0.539
							
**Percent**	**Percent**	**Proportion**		**Percent**	**Proportion**		
Sex (Female)	37.7%	23/61		37.3%	25/67	Chi^2	0.964
Side (Right)	47.5%	29/61		47.8%	32/67	Chi^2	0.980

**Table 2.** Overall pre- versus postoperative knee range of motion and patient reported outcomes.

	**Pre**			**Post**			
**Variable**	**Mean**	**SD**		**Mean**	**SD**	**Test**	**P**
Knee Extension	6.47	3.62		4.57	3.37	Paired t-Test	**<0.0001**
Knee Flexion	108.90	9.55		110.10	10.09	Paired t-Test	0.27
WOMAC	0.55	0.17		0.31	0.19	Paired t-Test	**<0.0001**
Pain	6.58	2.04		2.27	1.72	Paired t-Test	**<0.0001**
							
**Variable**	**Median**	**IQR**		**Median**	**IQR**	**Test**	**P**
UCLA	4.00	3-5		4.00	3-5	WSR	0.06
Oxford	36.00	31-40.25		0.00	21-33.25	WSR	**<0.0001**

WSR = Wilcoxon Signed-Rank test

**Table 3.** Comparison of postoperative patient reported outcomes between supine and lateral sleeping position.

		**Supine**			**Lateral**			
**Variable**		**Median**	**IQR**		**Median**	**IQR**	**Test**	**P**
Womac								
	Pre	0.56	0.14		0.54	0.20	t-Test	0.720
	Post	0.30	0.20		0.32	0.20	t-Test	0.596
Pain								
	Pre	6.74	1.86		6.57	2.07	t-Test	0.654
	Post	2.19	1.84		2.37	1.60	t-Test	0.584
								
**Variable**		**Mean**	**SD**		**Mean**	**SD**	**Test**	**P**
UCLA								
	Pre	4.00	6-3		4.00	5-3	Mann-Whitney	0.896
	Post	4.00	4-3		4.00	5-3	Mann-Whitney	0.424
Oxford								
	Pre	35.00	40.25-26		36.00	41.5-30.25	Mann-Whitney	0.260
	Post	28.00	32.5-18		27.00	34-21.75	Mann-Whitney	0.377

## Discussion

This is the first published report of data regarding the effects of sleeping position on terminal knee extension in primary TKA patients. This study demonstrated that individuals who reported to sleep predominantly in the supine position achieved significantly more knee extension at one month postoperatively in comparison to those that reported to sleep in the lateral recumbent position. Additionally, supine sleepers achieved significantly more knee extension when compared to their pre-operative measurements. In the postoperative measurements, the patients who slept predominantly in the supine position measured an average of 3 degrees of flexion contractures whereas the patients who slept predominantly in the lateral position measured an average of 6 degrees of flexion contracture. While these differences may seem relatively small, they may have a large clinical impact. Postoperative knee extension ROM is particularly important because patients with as little as 5 degree flexion contractures can have impaired biomechanics, gait and significant pain and dissatisfaction following TKA.^11^ Knowing this, surgeons generally prefer to implement physical therapy early in the postoperative period to improve and restore ROM^20^; however, those that fail physical therapy will likely undergo either lysis of adhesions and/or manipulation under anesthesia.^21^ Restoration of knee extension in the early postoperative period has been shown to limit the development of flexion contracture and is imperative to patient satisfaction following TKA.

The incidence of flexion contracture after primary TKA is between 1.4% and 17%.^6–9^ Mitsuyasu et al. concluded that contractures greater than 15 degrees at 3 months post-TKA would be unlikely to improve to less than 5 degrees at 2 years postoperatively. Those that do not improve to less than 5 degrees will continue to be sources of pain and abnormal gait kinematics leading to a dissatisfied patient.^22^ In this study, on average, patients in the supine sleeping position group had significantly greater knee extension than patients in the lateral sleeping position group. Patients who slept in the lateral position lacked on average 6 degrees of full extension which exceeds the 5 degree threshold for normal gait kinematics as described by Magit et al.^11^ The patients who slept in the supine position did not exceed the 5 degree threshold and therefore had normal gait biomechanics in relation to knee extension. Additionally, Kornuijt et al. found that patients with limited knee ROM at one month postoperatively demonstrated almost no improvement from weeks 4-8 in knee flexion and improvement in knee extension fluctuated during this time and even worsened during weeks 7-8.^23^ They also found that patients regained the greatest amount of knee extension in the first two weeks postoperatively.^23^ While this study only had one month follow up, the patients that reported to sleep in the lateral recumbent position showed a statistically significant decrease in knee extension at this time point, which based on the findings of Kornuijt et al. suggests that these patients would likely have limited improvement in comparison to patients with greater overall range of motion at one month.^23^ However, despite the statistically significant difference in postoperative knee flexion contracture between the supine and lateral groups, there was no difference in patient reported outcomes (WOMAC, pain, UCLA, Oxford). The lack of difference in patient reported outcomes may be due to the temporal proximity to the patient’s surgery and a difference would be seen at a later time point, the patient reported outcome measurements aren’t sensitive enough to detect a difference, or the difference in flexion contracture is not enough to produce a clinically significant effect. Whether altering sleep position can decrease the risk of knee flexion contractures following TKA cannot be answered by this study alone; however, this does warrant further investigation of the effects of sleeping position on knee ROM following TKA and suggests that patients who sleep in the lateral recumbent sleeping position may have the potential for worse outcomes than patients who sleep in the supine position.

There are several limitations to our study. As this was a non-randomized study, the observed results should be interpreted with caution. Patient sleeping position was a subjective response based on how the patient spent the majority of their time sleeping in the initial postoperative period and was not controlled for. Furthermore, a small cohort of patients, a single institution, and one surgeon, limit the generalizability of our findings. We only obtained measurements at one month follow-up postoperatively and therefore are unable to determine if the difference in knee extension was maintained through longer term follow-up. We were not able to fully ensure there were no confounding variables between groups in terms of outpatient physical therapy (e.g., how often, the patient’s dedication/compliance, and intensity). Furthermore, the administration of physical therapy exercises that our patients received what not standardized across all physical therapy clinics.

## Conclusions

In summary, our results demonstrate that sleeping position may affect initial postoperative knee range of motion. This is demonstrated by the statistically significant difference in knee extension between the supine and lateral sleeping position groups. The lateral sleeping position patients demonstrated on average a lack of 6 degrees of full extension which places them above the 5-degree threshold for normal gait mechanics. However, despite the significant difference in postoperative knee flexion contracture measurements, there was no difference in postoperative patient reported outcome measures. While the follow-up in this study is limited, it does identify lateral sleeping position as an easily identifiable and potentially preventable risk factor for flexion contracture that could help improve outcomes following primary TKA. We believe a larger, prospective, randomized controlled trial with longer term follow-up is warranted to confirm these findings. Our findings support recommending postoperative total knee arthroplasty patients sleep in a supine position to help avoid knee flexion contractures.

### Ethical Statement

We confirm that Institutional Review Board approval was obtained prior to conducting research. Informed consent was obtained from all patients involved in this study. Declarations of interest: none.

### Conflict of Interests

The authors declare that they have no known competing financial interests or personal relationships that could have appeared to influence the work reported in this paper.
